# Effect of malaria training on community healthcare providers’ compliance with malaria treatment guidelines in Nigeria using propensity score matching

**DOI:** 10.1186/s12936-026-05991-0

**Published:** 2026-06-09

**Authors:** Sikiru Baruwa, Toyin O. Akomolafe, Emeka Emmanuel Okafor, Osimhen Ubuane, Rodio Diallo, Michael Alagbile, Jennifer Ladokun, Johnson Ekele, Dayyabu Yusuf

**Affiliations:** 1 Population Council, Abuja, Nigeria; 2 Society for Family Health, Abuja, Nigeria; 3 Gates Foundation, Seattle, USA

**Keywords:** Community Pharmacists, Patent and proprietary medicine vendors, Malaria, Knowledge, Acceptability, Compliance, Propensity score matching, Nigeria

## Abstract

**Introduction:**

Community Pharmacists (CPs) and Patent and Proprietary Medicine Vendors (PPMVs) are important primary sources of care for many Nigerians. They are the first line of contact for those seeking advice or treatment for fever. However, the process needs to be strengthened and aligned with the World Health Organization (WHO) malaria treatment guidelines, as these providers currently diagnose based solely on symptoms. The IntegratE Project 2.0 is piloting innovative models to expand quality family planning and primary health care services through CPs and PPMVs across 11 Nigerian states, while generating evidence for policy reform. Our evaluation examines the effect of the malaria training module on knowledge, acceptability, and compliance with WHO treatment guidelines and identification of quality drugs among CPs and PPMVs, using a propensity score matching (PSM) analysis.

**Methods:**

A quasi-experimental evaluation design with a comparison group was implemented. Baseline and follow-up data were collected from February 2022 to December 2022 using structured questionnaires from self-selected intervention and randomly selected comparison groups. Descriptive statistics and PSM models were computed to estimate the average treatment effect (ATE) and average treatment effect for the treated (ATET). All analyses were performed using Stata 16.1, and a p-value < 0.05 was deemed significant.

**Results:**

Findings showed a significant positive impact of training on knowledge of malaria diagnosis and treatment (β = 0.052; 95% CI 0.006, 0.097; p = 0.03) and acceptability of the WHO test, treat, and refer guidelines among trained CPs and PPMVs (β = 0.432; 95% CI: 0.041, 0.822; p = 0.03). However, the training has no effect on compliance with treatment guidelines and identification of quality drugs.

**Conclusion:**

Malaria training using standard treatment guidelines modestly increases private providers’ knowledge and acceptance. A scale-up of malaria training for CPs and PPMVs is recommended, and further research is needed on guideline compliance.

**Supplementary Information:**

The online version contains supplementary material available at 10.1186/s12936-026-05991-0.

## Introduction

The World Health Organization (WHO) estimates that about 263 million cases of malaria occurred worldwide [1]. 26 out of 49 countries in sub-Saharan Africa accounted for almost 93% of the global malaria burden. The burden of malaria affects children under the age of five and pregnant women, resulting in high morbidity and mortality rates [1, 2]. It is reported that approximately 67% (272,000) of global malaria deaths are among children under five years of age [2]. The primary reason for this difference is that infants and young children under 5 years of age are at high risk of clinical episodes due to their underdeveloped immune systems against this infection [3–5]. Previous studies have reported that children under 2 years of age [6–11] are most susceptible to malaria infection. The consequences of malaria in children under five years of age include chronic anemia, low birth weight, impaired growth, and severe intellectual disabilities [12–14].

Nigeria accounts for a significant percentage of global malaria cases and deaths, contributing approximately 26% of all malaria cases, 31% of all malaria-related deaths worldwide, and 39% of malaria-related under-five mortality [1, 2]. An estimated 68 million malaria cases occurred in 2023 and nearly 165,000 malaria-related childhood deaths occur same year in Nigeria [1, 14].

To address this global and national concern, WHO in 2012 introduced a comprehensive malaria treatment guideline known as “test, treat, and refer,” to encompass malaria diagnosis, treatment, and referral [15]. This guideline demands confirming malaria cases through diagnostic testing before initiating treatment, tracking cases to ensure appropriate follow-up, and treating confirmed cases with the recommended antimalarial medications [15]. This policy or guideline is to replace the traditional focus, which was solely on treating malaria, to prioritize accurate diagnosis of the actual cause of fever. Effective evaluation of the malaria treatment guideline/policy stressed the important role of proper stock management of rapid diagnostic tests (RDTs) and laboratory supplies, coupled with comprehensive training and supervision, in ensuring high testing rates for suspected malaria cases [16–20]. This test before the treatment of the malaria approach [1, 15] appeared to have put malaria control on a front burner compared to previous approaches [21].

The dependence on clinical suspicion and microscopy for malaria diagnosis prior to the advent of rapid diagnostic tests (RDTs) often led to misdiagnosis and unnecessary use of antimalarials [21]. In Nigeria, blood smear malaria microscopy is limited at low-level and peripheral health care facilities because of a lack of the required skilled manpower, accessories, and reagents, high cost, and irregular electricity supply, among others [22]. Rapid diagnostic tests (RDTs), however, offer advantages such as accessibility, ease of use, and high diagnostic accuracy, addressing some of the challenges faced in resource-limited settings like Nigeria [21–25].

The use of RDTs to guide malaria treatment in routine care decisions among informal sector health practitioners in sub-Saharan Africa has the potential to reduce the seeming misdiagnosis and presumptive malaria treatment in this sector [26]. For example, Nigeria's national malaria control programs recognized the importance of community involvement and training initiatives for healthcare providers. The new malaria treatment guidelines mandated community-based providers, CPs, and PPMVs to test using RDTs during the treatment of malaria illnesses [27]. This proactive stance aims to enhance malaria case management, boost community engagement in preventive measures, and improve access to and quality of healthcare services at the grassroots level.

Adherence to the malaria treatment guidelines, especially among healthcare providers at the community level such as CPs and PPMVs, is a key challenge [19]. Most clients with suspected malaria are most likely to consult these healthcare providers first before referrals to hospitals or clinics. In situations where logistics/commodities are available, data have shown a lack of routine parasitological confirmation by PPMVs [28, 29]. Therefore, the crucial question remains: Will CPs and PPMVs adhere to the guidelines outlined in the national policy on malaria treatment in Nigeria? The objective of this study is to assess the effect of training and supervision on the adherence of CPs and PPMVs to the national policy on malaria treatment and assess their knowledge of the quality of drugs.

## Methods

### Intervention

The IntegratE project phase 2, funded by the Gates Foundation, is being implemented in 11 states in Nigeria. In collaboration with the Pharmacy Council of Nigeria (PCN), the IntegratE Project aims to increase access to family planning and PHC services in underserved areas through the involvement of the capacity of private sector providers, CPs, and PPMVs. The project currently covers eleven (11) states in Nigeria and, in addition to family planning, layered a modicum of primary health care services. A core delivery vehicle for the implementation of family planning services is adopting the PCN three-tier approach. Pharmacy Council of Nigeria (PCN) classified PPMVs into three groups or tiers, and different scopes of family planning training were provided based on their health qualifications. The three tiers are Tier 1 (non-health trained), Tier 2 (health trained), and Tier 3 (pharmacy technicians). A detailed description of the three-tier accreditation system of the IntegratE Project, the categories of PPMVs, and the type of training received can be found elsewhere [30, 31].

Under the IntegratE Project, CPs and PPMVs were trained on malaria diagnosis treatment and management as part of the Integrated Community Case Management (ICCM) training for children under 5. The malaria training was delivered in a classroom setting over a four-day period as part of the Mandatory Entry-Point Training Program (MEPTP), facilitated by the Schools of Health Technology [32]. The training was designed in accordance with the WHO guidelines for malaria case management. A key component of the training was the "Test, Treat, Refer" strategy, with a particular focus on children under five years of age [33]. This approach emphasizes the importance of testing using RDTs before initiating treatment. In cases involving children under five who test positive or exhibit severe symptoms, immediate treatment is administered, followed by referral to higher-level health facilities to ensure comprehensive care and reduce the risk of complications [33].

### Study design and setting

This study was part of a larger project, a quasi-experimental longitudinal study aimed at assessing knowledge retention, perception, and practices of PHC service delivery among CPs and PPMVs in intervention and comparison groups. In this study, endline data was used to evaluate the effects of the malaria training module on knowledge, acceptability, and compliance with WHO treatment guidelines, and identification of quality drugs among CPs and PPMVs, using a propensity score matching analysis to account for baseline differences. This study was conducted in eight (8) states: Enugu, Gombe, Kaduna, Kano, Lagos, Nasarawa, Niger, and Sokoto. Malaria prevalence varies across these states, with Kaduna, Kano, and Enugu states reporting high malaria prevalence, particularly among children under 5 years, and most northern states experience seasonal variations with malaria prevalence peaks during the wet season, especially in Kaduna and Kano [34]. In these states, private sector involvement is high, and both CPs and PPMVs are common and widely used. Patent and Proprietary Medicine Vendors (PPMVs) are trusted for quick treatment, and presumptive treatment is more entrenched [35, 36]. Baseline and endline data were collected between February 2022 and March 2023.

### Study participants and sampling technique

The sampling approach incorporated both non-random self-selection and random sampling to allocate participants to the intervention and comparison groups, respectively. Participants in the intervention group were recruited through non‑random self‑selection, because participation in the intervention was voluntary and routine program enrolment, not researcher‑assigned. To construct a comparison group, eligible non‑participants were selected using random sampling from a waiting list of the same population. Given the non‑random assignment to the intervention, propensity score matching was applied to adjust for observable baseline differences between the two groups and to approximate a counterfactual comparison. Pre- and post-training assessments were conducted for all trained CPs and PPMVs enrolled in the IntegratE PHC training. 816 CPs and PPMVs in the intervention states were self-selected into the study across five states; recruited through their state associations (Nigerian Association of Patent and Proprietary Medicine Dealers (NAPPMED) and Association of Community Pharmacists in Nigeria (ACPN)); encouraged to register with the PCN; and have expressed interest in participating in the PHC training. The 816 CPs and PPMVs who were enrolled in the intervention states (Gombe, Kaduna, Kano, Lagos, and Nasarawa) depends on the states and overall number of CPs and PPMVs trained. The comparison group of 302 CPs and PPMVs was drawn from a waiting list of individuals in three other states (Enugu, Niger, and Sokoto) that were yet to be trained by the IntegratE project in PHC services, using simple random sampling. 1,118 CPs and PPMVs who were assessed at baseline from eight states in Nigeria were included in this study.

The non-randomized nature of group allocation across the full sample limits causal inference. To mitigate this, baseline characteristics were assessed for both groups, and analytic adjustments using propensity score matching were conducted to account for potential confounding.

The study included a total sample of 1,118 CPs and PPMVs across eight states. State‑level stratification was applied to allocate the overall sample proportionally according to the distribution of eligible providers within each state. A complete sampling frame was compiled for all states to support proportional allocation. In the five intervention states, between 5 and 10% of eligible CPs and PPMVs were invited to participate, and enrolment was based on self‑selection among those invited. In the three control states, a simple random sampling approach was used to select 302 participants from a total population of 5,158 CPs and PPMVs, using computer‑generated random numbers. The overall sample size of 1,118 was derived using a single‑population proportion formula assuming a 95% confidence level, 5% margin of error, and a conservative proportion of 50%.

$$n_{0} = Z^{2} p{{\left( {1 - p} \right)} \mathord{\left/ {\vphantom {{\left( {1 - p} \right)} {d^{2} }}} \right. \kern-0pt} {d^{2} }}$$

where:

n_0_ = initial sample size

Z = Z‑score for 95% confidence level = 1.96

p = assumed population proportion

d = margin of error = 0.05

This yielded a base sample size of 384. To account for state‑level stratification, clustering of providers, and non‑random enrolment in intervention states, a design effect of 2.9 was applied, increasing the sample to 1,114. A further 5% adjustment for non‑response was made, yielding a target sample of approximately 1,170. The final achieved sample was 1,118 participants, of whom 73% were in the intervention group and 27% in the control group.

### Data collection procedures

A structured questionnaire was developed after a thorough review of relevant literature [37–40]. It was designed to capture information on provider knowledge, practices, and treatment compliance related to malaria case management. The questionnaire was digitized and deployed using the OpenDataKit (ODK) platform. The questionnaire was pre-programmed with skip logic to minimize data entry errors. Links were generated and distributed electronically to respondents, allowing them to complete the survey on their smartphones or tablets at their convenience. The use of ODK enabled real-time monitoring of submissions and facilitated secure data storage on encrypted servers. Data was collected from intervention and comparison CPs and PPMVs using self-administered structured questionnaires. A team of trained research assistants was responsible for supporting participants on how to navigate and complete the questionnaire using the ODK interface, following up with participants via phone calls, text messages, or in-person visits where necessary to remind them, and providing real-time technical support to address queries related to navigation issues within the tool. Before the actual data collection began, the tool was piloted and amended accordingly.

Baseline data were collected from February to April 2022 across the different states before training. Training (lasting three weeks per training) took place across the intervention states in batches from February to April 2022. After 6 months of implementation and practice, providers were followed up to see the impact of the training. From October to December 2022, 6 months after the completion of the training of CPs and PPMVs, a follow-up survey was administered to assess knowledge retention, perception, and practices of PHC service delivery among both intervention and comparison groups. 1,118 CPs and PPMVs enrolled in the study who completed the baseline and follow-up survey during and after the training, and the comparison group, who received no training but administered the baseline questionnaires, completed the self-administered follow-up questionnaire. The inclusion criteria for the intervention group include being 18 years or older, enrollment in the IntegratE 2.0 Project, and completion of training offered by the IntegratE project. For the comparison group: 18 years or older, received no training from the IntegratE project, CP/PPMVs providing PHC services, and finally similar to the accredited PPMV outlets in terms of location and type (i.e., health-trained and non-health-trained). All participants enrolled in the study were given detailed information about the study before consenting to participate.

### Measurement

Four key outcome measures were assessed in this study, with their detailed characteristics described below.

Knowledge of malaria was measured by a binary variable for whether the provider has sufficient knowledge of malaria diagnosis and treatment. This was created by assessing whether a healthcare provider (CPs and PPMVs) demonstrates sufficient knowledge on key aspects of malaria management by evaluating their responses to 30 questions related to diagnosis, recommended treatments, and understanding of malaria transmission. A score above a predefined threshold (≥ 14) indicates sufficient knowledge, coded as "1," while a score below 14 indicates insufficient knowledge, coded as "0." The composite score was dichotomized by assigning a fixed number of points to providers whose performance exceeded a predefined threshold [41].

The acceptability of the test, treat, and refer practices of malaria treatment was measured by using a composite score computed for each respondent by summing up the 9-item scale. This was used to assess how well CPs and PPMVs accept and support malaria testing, treatment, and referral practices by asking individuals about their perceptions, attitudes, beliefs, and willingness to engage with these practices. The response options for each attitudinal statement were ‘strongly agree,’ ‘agree,’ ‘strongly disagree,’ and ‘disagree.’ These were dichotomized into two categories: agree and disagree. Responses that aligned with the WHO test, treat, and refer guidelines were classified as correct (coded as 1), while those that did not were classified as incorrect (coded as 0).

Compliance with malaria treatment guidelines was operationalized as the total number of mRDT-confirmed malaria cases that were correctly treated with Artemisinin-based Combination Therapy (ACT). Compliance was assessed by comparing the number of malaria cases diagnosed using mRDTs (specifically those testing positive) with the number of cases treated with ACT. For each participating outlet, records were reviewed to determine the number of suspected malaria cases tested using mRDTs, the number of mRDT-positive cases, and the number of mRDT-positive cases treated with ACT. Providers who reported the number of malaria cases treated to be less than the number of positive cases were assumed to be non-compliant with protocol.

To assess providers’ ability to recognize quality-assured antimalarial medicines, we computed a composite score indicating whether a provider demonstrated sufficient knowledge of drug quality standards. Respondents were asked to identify the key features used to verify the quality of a medicine, particularly ACTs. Providers were expected to correctly mention all the following essential criteria: National Agency for Food and Drug Administration and Control (NAFDAC), drug label information (including the drug name, dosage strength, manufacturer's information, and expiry date), active ingredients, and features of a defective drug. The composite score was computed using 12 items.

The correlation values and Cronbach’s alpha coefficients for all items assessing knowledge, acceptability, and awareness of drug quality are presented in the Supplementary Tables (Additional files). Cronbach’s alpha values range from 0.60 to 0.70, indicating a moderate and acceptable level of internal consistency across measures.

### Independent variables

Data on individual characteristics of the study participants included: (1) age was recorded as a continuous variable; (2) sex was captured as male or female; (3) cadre was classified as Tier 1 (non-health-trained PPMV), Tier 2 (health-trained), Tier 3 (pharmacy technician), and CP (holds a degree in pharmacy); (4) marital status was classified as single, married/in a union, and widowed/divorced; (5) Level of education, that is, the highest level of formal education attained, was categorized as primary school or less, secondary, 2 + years post-secondary, and other professional certificate. (6) years in operation recorded as the total number of years the participant had been practicing in the pharmaceutical or drug retail sector as a continuous variable; (7) Ever worked in a health facility was dichotomized into yes or no; and (8) previous training in malaria case management, that is, whether the provider had received any formal training on malaria diagnosis and treatment of malaria in the past year, including government-led or donor-supported programs, was dichotomized into yes or no.

### Statistical analysis

The data collected by ODK was exported to Stata V.16.1, cleaned, edited, and analyzed. This study explored the effects of the intervention (training) on the four key outcome measures. Baseline characteristics of study participants were analyzed to identify the differences between the intervention and comparison groups prior to assessing intervention effects. Independent sample t-tests were conducted to evaluate the mean differences of participant characteristics across the two groups. The t-test was used for continuous variables and for binary and categorical variables that were coded as 0/1 dummy variables. All statistical tests were two-tailed, and significance was set at a p-value < 0.05. The results were presented as mean ± standard deviation for continuous variables or proportions for categorical variables.

### Propensity score matching (PSM)

To estimate the effect of the intervention on four key outcomes (provider knowledge, acceptability of the guidelines, compliance, and identification of quality drugs) while accounting for baseline differences between the intervention and comparison groups, we employed propensity score matching (PSM). This was used to reduce selection bias arising from the non-random assignment of participants to the groups, particularly since providers in the intervention group self-selected into the study.

### Estimation of propensity scores

Propensity scores, the conditional probability of a provider receiving the intervention given a set of observed covariates, were estimated using a logistic regression model. The model included a range of baseline covariates that could influence both the likelihood of receiving the intervention and the outcome variables, while controlling for observed baseline differences. The covariates entered into the model included treatment status, age, cadre, sex, level of education, marital status, years in operation, whether they had ever worked in a health facility, and previous training in malaria case management. The selection of covariates for the PSM was grounded in established causal inference frameworks and best practices in observational research design [42, 43]. The primary objective was to include pretreatment variables that predict both treatment assignments (i.e., participation in the intervention) and the outcome (provider knowledge), thereby reducing confounding bias and achieving covariate balance across treatment groups.

The nearest neighborhood matching method with matching of 1:3 among 1,118 CPs and PPMVs without replacement and a caliper of 0.1 was used to enhance precision by averaging across multiple matches. Only matched pairs within the specified caliper range were retained in the analysis, improving comparability between groups. The average treatment effect on the treated (ATET) was estimated using bias-corrected standard errors. A second model was used to determine the average treatment effect for the entire sample (ATE), and matching was not restricted based on proximity of propensity scores, thereby allowing a broader estimation of treatment effects across matched participants. This second model serves as a robustness check to confirm whether the treatment effect observed in the first model analysis holds under a less restrictive matching framework.

### Baseline covariate balance

To evaluate whether the matching procedure effectively reduced selection bias, we assessed baseline covariate balance between the intervention and comparison groups in the matched sample following the propensity score matching estimation. The observed baseline characteristics were considered to be well balanced after matching when the standardized mean differences (SMD) were less than 0.1 (or 10%). Large SMDs post-matching suggest residual imbalance and potential confounding.

### Assumptions

We examined the Conditional Independence Assumption (CIA) that underpins causal inference in observational studies to ensure the validity of the propensity score analysis. Also referred to as unconfoundedness, the CIA posits that after conditioning on observed covariates, treatment assignment is independent of potential outcomes. In other words, there are no unmeasured confounders. Covariates included in the propensity score model were carefully selected based on theoretical relevance and prior evidence linking them to both treatment assignment and the outcomes. Only baseline variables were included to avoid adjusting for mediators or variables affected by the intervention. This assumption is supported by covariates with SMDs < 0.1 post-matching, showing that they were considered adequately balanced.

### Ethical considerations

Ethical approval was obtained from the Population Council's Institutional Review Board (Protocol 878). Informed consent was obtained from each study participant prior to data collection and documented electronically. Consent was recorded electronically to ensure that the response is securely stored along with the survey data, reducing the risk of losing paper forms or mismatched records, and simplify data management. By requiring participants to actively select “Yes” before proceeding, the system enforces informed choice and prevents accidental participation without consent.

## Results

### Participants' characteristics

A total of 1,118 participants (CPs and PPMVs) were included in the analysis prior to matching, comprising both 816 intervention and 302 comparison groups. After applying PSM with a 1:3 nearest-neighbor algorithm and a caliper of 0.1, 1036 matched participants remained in the sample. Before PSM, an independent sample t-test was conducted to compare the proportion of participants between groups. There were statistically significant differences between groups in the distribution of Tier 2 PPMV, CP, sex, primary education, years in operation, ever having worked in a health facility, and previous training in malaria case management. The proportion of female providers was 39% in the intervention group and 24% in the comparison group. A lower proportion of CPs was observed in the intervention group (28%) compared to the comparison group (49%), while more Tier 2 PPMVs were represented in the intervention group. Participants in the intervention group reported more years in practice on average (11.3 years) than those in the comparison group (9.2 years) (Table 1). Several characteristics showed statistically significant differences between groups (p < 0.05), indicating potential selection bias and justifying the need for covariate adjustment using propensity score methods. After PSM analysis, all SMDs were substantially reduced, most falling well below the 0.1 (10%) threshold for acceptable balance between treatment and control groups.

**Table 1:** Mean characteristics of CPs and PPMVs at baseline, by group

Characteristics	Intervention (n = 818)	Control (n = 302)	Difference	p-value
Age (years)	38.8	39.7	0.9	0.10
Cadre (%)				
Tier 1	29.9	26.2	-3.7	0.22
Tier 2	34.2	19.9	-14.3*	0.00
Tier 3	7.7	4.6	-3.1	0.07
CP	28.2	49.3	21.2*	0.00
Sex (%)				
Male	60.7	75.5	14.8*	0.00
Female	39.3	24.5	-14.8*	0.00
Marital status (%)				
Not married	22.2	21.2	-0.9	0.72
Married/In union	77.8	78.8	0.9	0.72
Level of education (%)				
Primary school or less	0.2	5.6	5.4*	0.00
Secondary	19.0	14.9	-4.1	0.11
2 + years post-secondary	80.8	79.5	-1.3	0.63
Number of years in operation	11.3	9.2	-2.1*	0.00
Ever worked in a health facility (%)	69.9	57.0	-12.9*	0.00
Ever received malaria training (%)	77.2	92.1	14.8	0.00
Baseline outcomes (%)				
Knowledge of diagnosis and treatment of malaria	90.7	83.1	-7.6*	0.00

^*^Difference between groups significant at p < .05

### Acceptability of the test, treat, and refer practice for malaria

The acceptability of the test, treat, and refer practice for malaria among providers was assessed using nine attitudinal statements, as presented in Table 2. A significantly higher proportion of participants in the intervention group responded correctly to five out of the nine statements, compared to those in the comparison group. For instance, 80% of intervention participants correctly disagreed with the statement, *“Telling clients when to take their medication is less important if written instructions are provided,”* compared to 65% in the comparison group (p = 0.00). No statistically significant differences were observed between groups for two of the nine statements. On average, the mean number of correct responses (out of nine) was 7.0 (SD = 1.6) in the intervention group and 6.3 (SD = 1.6) in the comparison group, a difference that was also statistically significant (p = 0.00) (Table 2).

**Table 2:** CPs and PPMVs responses to nine attitudinal statements. (n = 779)

Statements	Correct response	% (95% CI) of participants’ providing correct response		p value
		Intervention (529)	Control (250)	
All clients who present with fever or suspected malaria should be tested for malaria infection by mRDT	Agree	87.1 (84.3, 90.0)	91.2 (87.7, 94.7)	0.10
Chloroquine is a recommended treatment for uncomplicated malaria infection	Disagree	68.8 (64.8, 72.8)	50.4 (44.2, 56.6)	0.00
In most cases, clinical diagnosis (physical examination) is just as accurate as mRDT in detecting malaria infection	Disagree	63.3 (59.2, 67.4)	42.8 (36.6, 49.0)	0.00
Fever clients who test negative for malaria infection should still be provided with antimalarial medication as a precautionary measure	Disagree	71.1 (67.2, 74.9)	47.6 (41.4, 53.8)	0.00
Telling clients when to take their medication is less important if written instructions are provided	Disagree	80.1 (76.7, 83.6)	65.2 (59.2, 71.1)	0.00
In most cases, artemisinin-based combination therapy is the most effective treatment for malaria infection	Agree	74.9 (71.1, 78.6)	83.2 (78.5, 87.9)	0.01
I will consider prescribing antimalarial drugs to a patient with negative mRDT if patient pressurizes me to prescribe it to them	Disagree	82.6 (79.4, 85.8)	64.8 (58.8, 70.8)	0.00
I do not often refer a client when I feel the case is beyond my capacity to handle	Disagree	83.5 (80.4, 86.7)	87.2 (83.0, 91.4)	0.19
It is important to refer clients with severe malaria infection	Agree	87.1 (84.3, 90.0)	97.2 (95.1, 99.3)	0.00
Overall Acceptability score (Mean)		7.0 (6.9, 7.1)	6.3 (6.1, 6.5)	0.00

### Compliance

Table 3 presents data on provider compliance with malaria case management protocols among CPs and PPMVs. A significantly higher proportion of patients in the intervention group received a malaria diagnostic test compared to the comparison group (68% vs. 53%). Among those who were tested, 80% of patients in the intervention group and 79% in the comparison group tested positive for malaria, a statistically significant difference, though the absolute difference was marginal. Despite diagnostic confirmation, not all positive cases received the recommended antimalarial treatment. Among confirmed malaria cases, 93% in the intervention group and 97% in the comparison group were prescribed appropriate antimalarial medications. Based on providers’ self-reported compliance with both testing and appropriate treatment protocols, compliance was significantly lower in the intervention group (79%) compared to the comparison group (86%; p = 0.01) (Table 4).

**Table 3:** CPs and PPMVs compliance with test and treatment of malaria

	Intervention	% (95% CI)	Control	% (95% CI)
	N		N	
Fever or suspected malaria cases seen at CP/PPMV premises	7529		4383	
Febrile patients tested for malaria infection using mRDT	5093	67.6 (66.6, 68.7)	2334	53.2 (51.8, 54.7)
Confirmed malaria cases (tested positive)	4070	79.9 (78.8, 81.0)	1843	79.0 (77.2, 80.6)
Confirmed malaria cases prescribed recommended antimalarial/s (Compliance)	3782	92.9 (92.1, 93.7)	1797	97.5 (96.7, 98.2)

**Table 4:** Follow-up Outcomes (Ttest)

Follow-up outcomes	Intervention (n = 529)	Control (n = 250)	Difference	p-value
Health workers who reported compliance (%)*	79.0	86.4	7.4	0.01

^*^ In this calculation, providers who reported number of treated malaria cases less than number of positive cases are assumed to be non-compliant with protocol)

### Knowledge of drug quality

Majority of intervention and comparison groups had fully correct knowledge about what constitutes a good-quality drug according to the given criteria (Table 5). The difference between the groups was significant: 84% in the intervention group and 96% in the comparison group. Almost all providers in both groups (81% intervention and 89% comparison) knew what a correct drug label is, including the expiry date of drugs and active ingredients indicated on the label. On average, the mean quality score (out of twelve) was 11.0 (SD = 1.0) in the intervention group and 11.4 (SD = 1.6) in the comparison group, a difference that was also statistically significant (p = 0.00).

**Table 5:** Knowledge of drug quality

	Intervention (529)	Control (250)	p value
All drugs should be registered with the National Agency for Food and Drug Administration and Control (NAFDAC)	98.9 (98.0, 99.8)	99.2 (98.1, 100.0)	0.67
A good quality drug contains: Right amount of active ingredients as mentioned on the label, within the accepted standard limits	84.3 (81.2, 87.4)	96.4 (94.0, 98.7)	0.00
A correct drug label should have a label that includes name and strength of the drug, lot number, expiry date, instructions for use and the manufacturer’s address	81.1 (77.7, 84.4)	89.2 (85.3, 93.0)	0.00
The quality of antimalaria drug can be verified using Mobile Authentication Service (MAS)	89.0 (86.4, 91.7)	99.6 (98.8, 100.0)	0.00
A defective drug can be identified using the following:			
Discolouration	96.0 (94.4, 97.7)	96.4 (94.1, 98.7)	0.80
Spotting	93.6 (91.5, 95.7)	91.2 (87.7, 94.7)	0.23
Unusual odour	92.1 (89.7, 94.4)	95.6 (93.0, 98.2)	0.07
Swollen blister pack	93.0 (90.8, 95.2)	95.6 (93.0, 98.2)	0.16
Broken seal and leakages	94.5 (92.6, 96.5)	96.4 (94.1, 98.7)	0.25
Caked powder, caked suspension	93.2 (91.0, 95.3)	94.8 (92.0, 97.6)	0.39
Particle in clear solution	93.6 (91.5, 95.7)	93.2 (90.1, 96.3)	0.84
Drug misspelt on package	89.6 (87.0, 92.2)	90.8 (87.2, 94.4)	0.60
Overall quality score (mean)	11.0 (10.9, 11.1)	11.4 (11.3, 11.5)	0.00

### Effect of malaria training on knowledge, acceptability, compliance and awareness of drug quality

After matching intervention and comparison groups, the average treatment effect of malaria training on knowledge of diagnosis and treatment of malaria among the treated group (ATET) was 5.2% (β = 0.052; 95% CI: 0.006, 0.097; p = 0.03). A robust check to confirm whether the treatment effect observed in the more conservative primary analysis held under a less restrictive matching framework was carried out. The ATE in the population was computed while omitting the caliper restriction; the probability of knowledge was 5.7% (β = 0.057; 95% CI: 0.015, 0.099; p = 0.01) higher among intervention participants who received training compared to the comparison group (Table 6).

**Table 6:** Propensity score matching analysis on effect of malaria training on knowledge, acceptability and compliance with WHO treatment guidelines, and identification of quality drugs

Effect of malaria training on	ATE (95%CI)	p—value	ATET (95%CI)	p—value
Knowledge of malaria diagnosis and treatment	0. 057 (0.015, 0.099)	**0.01**	0.052 (0.006, 0.097)	**0.03**
Acceptability of the WHO test, treat and refer guidelines	0.562 (0.242, 0.883)	**0.00**	0.432 (0.041, 0.822)	**0.03**
Compliance with WHO treatment guidelines	− 0.063 (− 0.129, 0.003)	0.06	− 0.071 (− 0.144, 0.002)	0.06
Knowledge of drug quality	0.562 (0.242, 0.883)	**0.00**	− 0.235 (− 0.498, 0.027)	0.08

*ATE* Average treatment effect, *ATET* Average treatment effect on the treated

The estimated ATET of the malaria training on the acceptability of the WHO test, treat, and refer guidelines was 43.2% (β = 0.432; 95% CI: 0.041, 0.822; p = 0.03). The ATE across the population was 56.2% (β = 0.562; 95% CI: 0.242, 0.883; p = 0.00) (Table 6).

After matching, there was no statistically significant impact of the malaria training on compliance with WHO treatment guidelines between the intervention and comparison groups. The estimated ATET was − 7.1% (β = − 0.071; 95% CI: − 0.144, 0.002; p = 0.06), and the ATE for the overall population was -6.3% (β = -0.063; 95% CI: − 0.129, 0.003; p = 0.06) (Table 6).

Our results indicate that knowledge of drug quality would increase by 56% if all providers (CPs and PPMVs) in both groups received malaria training (ATE, β = 0.562; 95% CI: 0.242, 0.883; p = 0.00; see Table 6). However, the ATET was − 23.5% (β = − 0.235; 95% CI: − 0.498, 0.027; p = 0.08) and not statistically significant, suggesting no observable impact among those who actually received the training.

## Discussion

This study assessed the effect of the malaria training module on knowledge, acceptability, and compliance with WHO treatment guidelines and identification of quality drugs among CPs and PPMVs, using a propensity score matching analysis. The WHO treatment guideline recommends that every suspected malaria case should be tested; in other words, malaria should be diagnosed either by microscopy or malaria RDT before treatment with artemisinin combination therapy as per in-country guidelines.

First, we find that training on malaria modules has significantly improved knowledge of malaria treatment guidelines among CPs and PPMVs that received the training intervention compared to those that are not in the intervention. This includes increased knowledge of malaria transmission, symptoms, and appropriate treatment protocols, such as the use of ACTs. This effect is supported by a Nigerian study showing improved knowledge and practices [44] among trained PPMVs and a Sub-Saharan Africa meta-analysis highlighting the effectiveness of health education in improving malaria knowledge and practice [45]. Training that reinforces knowledge of the National Malaria Treatment Guidelines is therefore critical for knowledge attainment and improvement over time for the health practitioners in general. Regular and continuous training is vital to ensure that the knowledge of health practitioners, including CPs and PPMVs, remains up-to-date and relevant overtime [46].

Second, this study revealed that the CPs and PPMVs that received the malaria training module had a higher level of acceptability (their acceptability of the test, treat, and refer guidelines increased by an average of 43%) of the malaria treatment guideline as compared to CPs and PPMVs that did not receive the malaria training. There was a significant improvement in positive attitude regarding malaria treatment guideline post-training intervention, implying the potential sustainability of the intervention. A positive attitude is necessary and critical to ensure that interventions work and people accept, trust, and live based on the information and perception they have [47]. This indicates that targeted training modules effectively increase the willingness of CPs and PPMVs to adopt and implement the standard malaria treatment protocols, potentially improving the quality of malaria management services in the community. According to this study, training greatly increased CPs' and PPMVs' acceptance of the WHO malaria standards. The average increase in acceptability was a substantial 43%. The study demonstrates that providing malaria training modules is a viable strategy to improve adherence to malaria treatment guidelines by private healthcare providers.

Third, this study revealed that malaria training had no meaningful impact on improving compliance with WHO treatment guidelines, and in fact, a small negative trend was observed. In other words, training was not a statistically significant factor associated with adherence to the malaria treatment guidelines. Findings from similar studies carried out in Nigeria showed inconsistent impacts of training on health workers’ adherence to test outcomes [48–50]. Complete compliance with the results of diagnostic tests was first associated with a study that involved community health worker training [48]. In a similar vein, throughout a 3-year intervention period, supervisory visits and health worker training reduced the prescription of ACTs for patients in Zamfara and Kano states who tested negative for mRDT [50]. On the other hand, Niger State's direct supervision of health workers and the use of cascade training did not improve test outcomes [49]. Remarkably, when it came to treating mRDT-negative children appropriately, the non-intervention group did significantly better than the intervention group (93% versus 37%) [49]. This disparity might have been caused by the intervention group's skepticism or lack of faith in mRDT-negative results.

This result reinforces the notion in previous studies that compliance goes beyond training. This study shows that being trained does not necessarily translate into correct diagnostic and treatment practices, and this is consistent with the results from other studies [51–53]. This sheds light on the fact that beyond training CPs and PPMVs, there are likely other determinants of malaria treatment that need to be addressed, as previous studies had confirmed [54–56]. For example, availability of antimalarial drugs was observed to be a major factor that affected treatment prescription in both public and private health settings. This prompted health workers to have no choice but to prescribe the only recommended antimalarial drugs available in the facilities. This supports the results of earlier studies that found that prescribing patterns are more likely to follow the availability of antimalarial drugs [57]. Project intervention should look into behavioral factors that may influence health workers’ malaria treatment prescriptions to improve the treatment of malaria in health facilities in the state.

Lastly, the study indicates malaria training potentially improves overall knowledge of drug quality, but surprisingly, those who received the training had less correct knowledge about good drug quality (84% vs. 96%) than the control group. This suggests a negative impact or lack of effect from the training itself on the "trained" intervention group regarding their knowledge of drug quality, despite the training potentially having a broad positive effect in general. While the data show strong potential for improving drug quality knowledge through malaria training (ATE = 56%), the lack of effect among those already trained (ATET = − 23.5%) signals a need to strengthen training implementation, target the right participants, or address barriers to learning uptake.

In general, evidence from this study and others indicates inadequate compliance among healthcare workers in Nigeria, despite undergoing malaria training. Further research will be necessary to comprehend the effectiveness of various training approaches in enhancing practices, including mentoring, prolonged classroom training, or alternative strategies designed to promote and facilitate adherence among community healthcare professionals. Moreover, additional details are required regarding other elements such as incentives that could impact ADR reporting in primary healthcare environments [58].

## Limitations

This study is not without limitations. Firstly, the intervention group were self-selected into the program, but this introduces potential self-selection bias, as participants may differ from non-participants in motivation or other unmeasured characteristics. Secondly, the data gathered were self-reported. This was, however, reduced by assuring the respondents prior to the interview that privacy would be preserved. Although the number of CPs and PPMVs examined and the self-selection bias in this study may limit its scope and generalization, the results however reflect the general pattern of practice in the Community sector, as they are very similar to those of previous studies carried out in the region [44, 46] and elsewhere [39, 41]. However, the study needs to be extended to support the conclusions on better policy interventions to improve the management of malaria cases in the sector.

## Conclusion

Although effect sizes were small, malaria training using WHO standard treatment guidelines increases private providers’ knowledge and their acceptability of the guidelines. A scale-up of malaria training for private providers, especially CPs and PPMVs, is strongly recommended to improve the quality of services provided. Compliance with WHO malaria treatment guidelines is beyond training alone as there are likely other determinants of malaria treatment that need to be addressed, as previous studies had confirmed [54–56].Further study is recommended to examine these factors affecting compliance with malaria treatment guidelines. This evaluation examined the effect of the training on relevant outcomes and provided tangible evidence that may inform course correction during project implementation.

## Supplementary Information

Additional file 1.

## Data Availability

The deidentified dataset analyzed during the current study will be available on the Population Council’s Dataverse, [10.7910/DVN/AGYTS4] (https:/doi.org/10.7910/DVN/AGYTS4). [59]. The data will also be available from the corresponding author upon reasonable request.

## Abbreviations

ACPN: Association of Community Pharmacists in Nigeria
ACT: Artemisinin-based Combination Therapy
ATE: Average treatment effect
ATET: Average treatment effect for the treated
CI: Confidence interval
CIA: Conditional Independence Assumption
CPs: Community Pharmacists
ICCM: Integrated Community Case Management
MEPTP: Mandatory Entry-Point Training Program
NAFDAC: National Agency for Food and Drug Administration and Control
NAPPMED: Nigerian Association of Patent and Proprietary Medicine Dealers
ODK: OpenDataKit
PCN: Pharmacy Council of Nigeria
PPMVs: Patent and Proprietary Medicine Vendors
PSM: Propensity score matching
RDTs: Rapid diagnostic tests
SMD: Standardized mean differences
WHO: World Health Organization
