# Supplementary material for: Effect of malaria training on community healthcare providers’ compliance with malaria treatment guidelines in Nigeria using propensity score matching
Source: Malar J. 2026 Jun 9;25:255. doi: 10.1186/s12936-026-05991-0 (PMC13330328; doi:10.1186/s12936-026-05991-0)
Supplement: Supplementary file 1 — Additional file 1. [file 12936_2026_5991_MOESM1_ESM.docx]

**Table S1. The values of correlation and Cronbach Alpha for the construct of knowledge of malaria diagnosis, and treatment.**

| **Item** | **item-test correlations** | **item-rest correlations** | **average interitem correlation** | **alpha** |
| --- | --- | --- | --- | --- |
| Fever is not the commonest symptom of malaria | 0.25 | 0.14 | 0.08 | 0.70 |
| Malaria may cause miscarriage and low birth weight in pregnancy. | 0.22 | 0.12 | 0.08 | 0.70 |
| Which group is most vulnerable to malaria? (children under five years of age) | 0.33 | 0.23 | 0.08 | 0.70 |
| Malaria cannot be caused by standing in the sun. | 0.34 | 0.24 | 0.08 | 0.69 |
| Use of Long-Lasting Insecticidal Nets is one of the ways of preventing malaria. | 0.17 | 0.07 | 0.08 | 0.71 |
| **How would you treat a child exhibiting signs of severe malaria?** |  |  |  |  |
| Do Malaria RDT test | 0.36 | 0.26 | 0.08 | 0.69 |
| Treat using anti-malarial drugs | 0.46 | 0.37 | 0.07 | 0.68 |
| Give paracetamol | 0.31 | 0.20 | 0.08 | 0.70 |
| Place tepid sponge on child's forehead, sides of the neck, under the armpits | 0.18 | 0.07 | 0.08 | 0.71 |
| Refer to health facility | 0.17 | 0.06 | 0.08 | 0.71 |
| **Danger signs of severe malaria in a child under the age of two** |  |  |  |  |
| Persistent fever | 0.19 | 0.08 | 0.08 | 0.71 |
| Severe vomiting | 0.22 | 0.12 | 0.08 | 0.70 |
| Convulsions | 0.33 | 0.23 | 0.08 | 0.70 |
| Inability to sit or stand | 0.23 | 0.13 | 0.08 | 0.70 |
| Inability to eat, drink or breastfeed | 0.24 | 0.14 | 0.08 | 0.70 |
| Coma | 0.29 | 0.19 | 0.08 | 0.70 |
| **Drugs recommended for treatment of uncomplicated malaria** |  |  |  |  |
| Artemether–lumefantrine | 0.04 | -0.06 | 0.09 | 0.72 |
| Artesunate–amodiaquine | 0.25 | 0.14 | 0.08 | 0.70 |
| Chloroquine (Incorrect) | 0.52 | 0.44 | 0.07 | 0.68 |
| Sulfadoxine–pyrimethamine (SP)(Incorrect) | 0.46 | 0.37 | 0.07 | 0.68 |
| Artesunate monotherapy | 0.53 | 0.45 | 0.07 | 0.68 |
| Artesunate injection (Incorrect) | 0.55 | 0.47 | 0.07 | 0.68 |
| Amodiaquine (Incorrect) | 0.56 | 0.48 | 0.07 | 0.68 |
| Artemether injection | 0.49 | 0.40 | 0.07 | 0.68 |
| Quinine | 0.51 | 0.42 | 0.07 | 0.68 |
| Herbal fever mixture | 0.44 | 0.35 | 0.07 | 0.69 |
| Antibiotic | 0.44 | 0.34 | 0.08 | 0.69 |
| Paracetamol | 0.21 | 0.10 | 0.08 | 0.70 |
| **Test scale = mean (standardized items)** |  |  | **0.08** | **0.70** |

**Table S2. The values of correlation and Cronbach Alpha for the construct of acceptability of the test, treat and refer practices of malaria treatment.**

| **Item** | **item-test correlations** | **item-rest correlations** | **average interitem correlation** | **alpha** |
| --- | --- | --- | --- | --- |
| All clients who present with fever or suspected malaria should be tested for malaria infection by mRDT (Agree) | 0.48 | 0.28 | 0.14 | 0.57 |
| In most cases, artemisinin-based combination therapy is the most effective treatment for malaria infection (Agree) | 0.40 | 0.18 | 0.16 | 0.60 |
| It is important to refer clients with severe malaria infection. (Agree) | 0.45 | 0.25 | 0.15 | 0.58 |
| Chloroquine is a recommended treatment for uncomplicated malaria infection (Disagree) | 0.49 | 0.29 | 0.14 | 0.57 |
| In most cases, clinical diagnosis (physical examination) is just as accurate as mRDT in detecting malaria infection (disagree) | 0.53 | 0.34 | 0.14 | 0.56 |
| Fever clients who test negative for malaria infection should still be provided with antimalarial medication as a precautionary measure (disagree) | 0.57 | 0.39 | 0.13 | 0.55 |
| Telling clients when to take their medication is less important if written instructions are provided (disagree) | 0.46 | 0.25 | 0.15 | 0.58 |
| I will consider prescribing antimalarial drugs to a patient with negative mRDT if patient pressurizes me to prescribe it to them (disagree) | 0.55 | 0.36 | 0.13 | 0.55 |
| I do not often refer a client when I feel the case is beyond my capacity to handle. | 0.46 | 0.25 | 0.15 | 0.58 |
| **Test scale = mean (standardized items)** |  |  | **0.14** | **0.60** |

**Table S3. The values of correlation and Cronbach Alpha for the construct of knowledge of quality drugs**

| **Item** | **item-test correlations** | **item-rest correlations** | **average interitem correlation** | **Alpha** |
| --- | --- | --- | --- | --- |
| All drugs should be registered with the National Agency for Food and Drug Administration and Control (NAFDAC) | 0.24 | 0.07 | 0.19 | 0.72 |
| A good quality drug contains: Right amount of active ingredients as mentioned on the label, within the accepted standard limits. | 0.37 | 0.21 | 0.17 | 0.70 |
| A correct drug label should have a label that includes name and strength of the drug, lot number, expiry date, instructions for use and the manufacturer’s address | 0.31 | 0.14 | 0.18 | 0.71 |
| The quality of antimalaria drug can be verified using Mobile Authentication Service (MAS) | 0.33 | 0.16 | 0.18 | 0.71 |
| **A defective drug can be identified using the following:** |  |  |  |  |
| Discolouration | 0.55 | 0.41 | 0.16 | 0.67 |
| Spotting | 0.56 | 0.43 | 0.15 | 0.67 |
| Unusual odor | 0.59 | 0.46 | 0.15 | 0.66 |
| Swollen blister pack | 0.59 | 0.46 | 0.15 | 0.66 |
| Broken seal and leakages | 0.64 | 0.52 | 0.15 | 0.65 |
| Caked powder, caked suspension | 0.58 | 0.45 | 0.15 | 0.66 |
| Particle in clear solution | 0.51 | 0.37 | 0.16 | 0.68 |
| Drug misspelt on package | 0.53 | 0.39 | 0.16 | 0.67 |
| **Test scale = mean (standardized items)** |  |  | **0.16** | **0.70** |
